# A Novel Saliva RT-LAMP Workflow for Rapid Identification of COVID-19 Cases and Restraining Viral Spread

**DOI:** 10.3390/diagnostics11081400

**Published:** 2021-08-03

**Authors:** Gerson Shigeru Kobayashi, Luciano Abreu Brito, Danielle de Paula Moreira, Angela May Suzuki, Gabriella Shih Ping Hsia, Lylyan Fragoso Pimentel, Ana Paula Barreto de Paiva, Carolina Regoli Dias, Naila Cristina Vilaça Lourenço, Beatriz Araujo Oliveira, Erika Regina Manuli, Marcelo Andreetta Corral, Natale Cavaçana, Miguel Mitne-Neto, Maria Mirtes Sales, Luiz Phellipe Dell’ Aquila, Alvaro Razuk Filho, Eduardo Fagundes Parrillo, Maria Cássia Mendes-Corrêa, Ester Cerdeira Sabino, Silvia Figueiredo Costa, Fabio Eudes Leal, Germán Gustavo Sgro, Chuck Shaker Farah, Mayana Zatz, Maria Rita Passos-Bueno

**Affiliations:** 1 Centro de Pesquisa Sobre o Genoma Humano e Células-Tronco (HUG-CELL), Instituto de Biociências, Universidade de São Paulo (USP), São Paulo 05508-090, Brazil; lucianoab00@gmail.com (L.A.B.); daniellepmoreira@gmail.com (D.d.P.M.); may.suzuki@gmail.com (A.M.S.); gabriella.hsia@gmail.com (G.S.P.H.); lylyan.pimentel@gmail.com (L.F.P.); ana.p.behebak@gmail.com (A.P.B.d.P.); carol.regoli@gmail.com (C.R.D.); lourenconcv@gmail.com (N.C.V.L.); marcelo.acorral@gmail.com (M.A.C.); natale@gmail.com (N.C.); mayazatz@usp.br (M.Z.); 2 Instituto de Medicina Tropical, Universidade de São Paulo (USP), São Paulo 05403-000, Brazil; bea.a.oliv0@gmail.com (B.A.O.); erikamanuli@gmail.com (E.R.M.); cassiamc@uol.com.br (M.C.M.-C.); sabinoec@gmail.com (E.C.S.); silviacosta@usp.br (S.F.C.); 3 Grupo Fleury, Research and Development, São Paulo 04344-070, Brazil; mmitne@gmail.com; 4 Instituto de Ensino e Pesquisa Prevent Senior, São Paulo 04547-100, Brazil; maria.mirtes@preventseniormedicinadiag.com.br (M.M.S.); luiz.aquila@institutopreventsenior.com.br (L.P.D.A.); alvaro.filho@preventsenior.com.br (A.R.F.); eduardo@preventsenior.com (E.F.P.); 5 Faculdade de Medicina, Universidade Municipal de São Caetano do Sul (USCS), São Paulo 09521-160, Brazil; fabio.leal@online.uscs.edu.br; 6 Instituto de Química, Universidade de São Paulo (USP), São Paulo 05508-000, Brazil; ggsgro@iq.usp.br (G.G.S.); chsfarah@iq.usp.br (C.S.F.); 7 Faculdade de Ciências Farmacêuticas de Ribeirão Preto, Universidade de São Paulo, Ribeirão Preto 14040-903, Brazil

**Keywords:** LAMP (loop-mediated isothermal amplification) assay, 2019 novel coronavirus, saliva, viral diagnostics

## Abstract

Rapid diagnostics is pivotal to curb SARS-CoV-2 transmission, and saliva has emerged as a practical alternative to naso/oropharyngeal (NOP) specimens. We aimed to develop a direct RT-LAMP (reverse transcription loop-mediated isothermal amplification) workflow for viral detection in saliva, and to provide more information regarding its potential in curbing COVID-19 transmission. Clinical and contrived specimens were used to optimize formulations and sample processing protocols. Salivary viral load was determined in symptomatic patients to evaluate the clinical performance of the test and to characterize saliva based on age, gender and time from onset of symptoms. Our workflow achieved an overall sensitivity of 77.2% (*n* = 90), with 93.2% sensitivity, 97% specificity, and 0.895 Kappa for specimens containing >10^2^ copies/μL (*n* = 77). Further analyses in saliva showed that viral load peaks in the first days of symptoms and decreases afterwards, and that viral load is ~10 times lower in females compared to males, and declines following symptom onset. NOP RT-PCR data did not yield relevant associations. This work suggests that saliva reflects the transmission dynamics better than NOP specimens, and reveals gender differences that may reflect higher transmission by males. This saliva RT-LAMP workflow can be applied to track viral spread and, to maximize detection, testing should be performed immediately after symptoms are presented, especially in females.

## 1. Introduction

Molecular diagnostics of the novel coronavirus (SARS-CoV-2) amidst the COVID-19 pandemic has been crucial for monitoring infection dynamics and preventing the spread of the disease. The gold standard has been Real-Time Polymerase Chain Reaction (RT-PCR), which is performed on nasopharyngeal and oropharyngeal (NOP) specimens that pose discomfort to patients and require specialized materials and trained healthcare professionals for collection. In addition, RT-PCR typically requires viral inactivation followed by a lengthy RNA extraction/isolation step, further complicating diagnostic workflows and increasing the turnaround time for reporting results. Faster and simplified protocols for viral detection are desirable to curb transmission, especially in point-of-care settings and places that lack infrastructure or access to material or financial means. Reverse transcription loop-mediated isothermal amplification (RT-LAMP) has emerged as a viable, affordable alternative to RT-PCR, since it allows the rapid and direct detection of pathogens without nucleic acid extraction and sophisticated equipment [1].

Although SARS-CoV-2 is transmitted via infected saliva droplets and aerosols, attention has been focused on the upper airway tract rather than the oral cavity. Recently, viral shedding has been observed in the salivary glands and oral mucosa, further implicating saliva in infection and transmission [2]. Saliva has been considered a suitable alternative specimen for COVID-19 molecular diagnostics and to monitor viral spread, as it is easily accessible and can be collected by unsupervised patients into simple airtight vessels, diminishing costs and risk of transmission [3]. The implementation of salivary diagnostics must account for some issues, such as poor understanding of the viral biology in the oral cavity and viral load dynamics across individuals, which is important to determine the optimal test window to detect SARS-CoV-2. Furthermore, the development of robust protocols for viral detection in saliva may be useful for the diagnosis of other respiratory pathogens.

Here, we describe a novel workflow for RT-LAMP-based detection of SARS-CoV-2 that includes a stabilization solution to prepare saliva specimens without RNA isolation. This workflow stabilizes viral RNA, allows sample manipulation without biosafety rooms and cabins, and shows 93.2% sensitivity for viral loads above 10^2^ μL of saliva, 97% specificity, and 0.895 Kappa coefficient. We also provide insights into viral load differences between saliva and NOP swab specimens and relate them to gender, age, and time from onset of symptoms, further elucidating the diagnostic capabilities of saliva and bringing forth recommendations to maximize chances of detection. This rapid and efficient workflow is suitable for COVID-19 diagnostics in both centralized and point-of-care settings, and may be of particular value for use in places lacking sophisticated infrastructure.

## 2. Methods

### 2.1. Subjects

This project was approved by the Ethics Committee of Instituto de Biociências, Universidade de São Paulo, Brazil (accession number 31655320.0.0000.5464; 14 May 2020), and involved the collaboration with several groups in order to obtain anonymized clinical samples from individuals with respiratory symptoms.

Crude saliva samples from 26 symptomatic individuals were collected in August 2020 by two clinical laboratories (Instituto de Ensino e Pesquisa Prevent Senior and Grupo Fleury, both located in São Paulo, Brazil) and one research group (Instituto de Medicina Tropical, Universidade de São Paulo—IMT-USP, São Paulo, Brazil), and sent to our laboratory in ice on the same day. These individuals have been tested positive for SARS-CoV-2 via RT-PCR on nasopharyngeal/oropharyngeal (NOP) specimens by those institutions, and cycle threshold (Ct) values were shared whenever necessary.

Crude saliva and NOP samples from 131 symptomatic individuals were collected in January-February 2021 at Universidade Municipal de São Caetano do Sul/IMT-USP [4] (51 individuals for characterization of the diagnostic yield of saliva compared to NOP swabs—29 females and 22 males—and 80 for the blind study—48 females and 32 males). Saliva samples were aliquoted locally and transported to our laboratory in dry ice, while NOP specimens were tested for SARS-CoV-2 by RT-PCR in each institution, which shared Ct results and clinical data whenever necessary.

### 2.2. Saliva Collection

Briefly, individuals were asked not to eat for at least 30 min before collection of 3 mL of saliva in sterile, nuclease-free 15 mL conical tubes. Immediately post-collection, saliva was heat-inactivated by incubation at 95 °C.

### 2.3. DGS Preparation

We developed a solution (detailed information is described in the ‘Results’ section), named as ‘DGS’ (DTT/GuHCl solution), that prevents degradation of viral RNA and provides a better stabilization of the viral genome for RT-LAMP reactions. DGS contains 30 mM Tris-HCl pH 8.0, 600 mM GuHCl and 200 mM DTT, diluted in nuclease-free _dd_H_2_O. Inactivated saliva samples are mixed with this solution and incubated at 55 °C for 5 min prior to RT-LAMP reactions.

### 2.4. RT-LAMP and rtRT-LAMP Reactions

RT-LAMP reactions (12.5 μL total volume) contained 1x WarmStart^®^ Colorimetric LAMP Master Mix (New England Biolabs, Ipswich, MA, USA; #M1800L), a primer set composed of 1.6 μM FIP/BIP internal primers, 0.4 μM LF/LB loop primers and 0.2 μM F3/B3 external primers, and 1.25 μL of DGS:saliva mixture in 1:1 ratio, previously centrifuged at 1000× g for 30 s. Previously published primer sets targeting different regions of SARS-CoV-2 genome and the human gene ACTB were used (Table S1). All primer sets targeting SARS-CoV-2 have already been tested for specificity against other respiratory pathogens [5,6,7,8,9]. RT-LAMP reactions were carried out at 65 °C for 30 to 40 min.

For real time analysis of RT-LAMP (rtRT-LAMP), the above reaction was supplemented with 1 μM SYTO^®^-9 DNA binding dye (Thermo Fisher, #S34854) and 0.125 μL Low ROX reference dye (New England Biolabs, Ipswich, MA, USA; #E7638A), and incubated in a QuantStudio 5 qPCR machine (Thermo Fisher, Waltham, MA, USA) at 65 °C for 30 or 40 min (fluorescence signal acquisition at 15-s intervals), followed by a dissociation curve stage from 95 to 60 °C with temperature change rate of 0.1 °C/s.

To improve color discrimination, the reaction protocols above was further adjusted to contain 1.32x WarmStart^®^ Colorimetric LAMP Master Mix and 1 μL of DGS:saliva mixture in 2:1 ratio. Time to threshold (Tt) of positivity was defined at threshold = 0.8 relative fluorescence units. Each batch of reactions included positive controls with 1000 and 500 copies of SARS-CoV-2 RNA per reaction. Analysis of amplification plots and melting temperatures (Tm) were used to discriminate non-specific from specific amplifications (specific Tm = median Tm_positive control_ ± 1 °C).

### 2.5. RNA Isolation and RT-PCR Reactions on Saliva Samples

SARS-CoV-2 RNA was isolated from crude saliva samples using QIAamp Viral RNA Mini Kit (QIAGEN, Hilden, Germany; #52906), following manufacturer’s recommendations. RT-PCR was performed based on CDC’s protocol, which targets SARS-CoV-2 gene N (Centers for Disease Control and Prevention, 2020). RT-PCR reactions (12 μL total volume) contained TaqMan^®^ Fast Virus 1-Step Master Mix (Thermo Fisher, Waltham, MA, USA; #4444432) and a primer/probe mix (500 nM forward primer, 500 nM reverse primer, 125 nM probe; 2019-nCoV_N1, IDT, Coralville, IA, USA; #10006600), and were cycled in a QuantStudio 5 qPCR machine as per manufacturer’s recommendations (Thermo Fisher, Waltham, MA, USA). Absolute quantification of viral RNA copies was performed via standard curve assays with 2019-nCoV_N_Positive Control (IDT, Coralville, IA, USA; #10006625), in triplicates.

### 2.6. Simulation of Saliva Positive Controls with SARS-CoV-2 RNA

SARS-CoV-2 RNA was isolated from cell culture pellets (kindly provided by collaborators from Instituto de Ciências Biomédicas, USP), and viral titer was determined via absolute quantification with RT-PCR, as described above. Quantified SARS-CoV-2 RNA was spiked into saliva from a healthy donor (previously processed with 2:1 ratio of DGS: saliva and tested negative for SARS-CoV-2) to produce simulated specimens, and then stored in 10 µL aliquots at −80 °C for further use in RT-LAMP reactions.

### 2.7. Statistical Analyses

D’Agostino–Pearson normality test was applied to evaluate data distribution and to select appropriate statistical analyses to compare groups. Graphing and analyses were carried out with Graphpad Prism (v. 5.0.3). Statistical significance was set at *p* < 0.05.

## 3. Results

### 3.1. Stabilization of SARS-CoV-2 RNA in Saliva

Searching for a solution capable of stabilizing SARS-CoV-2 RNA in saliva, we initially screened six formulations containing Proteinase K (namely PK1-6). AVL, a commercial guanidine-based buffer recommended for SARS-CoV-2 inactivation [10], was used as the experimental control, without any heat treatment. Since unprotected viral RNA is rapidly degraded in crude saliva, we simulated samples by first mixing saliva from a healthy donor with the solutions (1:1, *v*/*v*) and heating at 65 °C for 15 min followed by a step at 95 °C for 2 min, before spiking in SARS-CoV-2 RNA. After sample processing, RNA was isolated and RT-PCR targeting the N gene was performed. We observed that PK6 led to similar Ct values to AVL (23.9 and 23.15, respectively), while no amplification was detected for the remaining PK formulations. Notably, PK6 and AVL were the only solutions that contained guanidine hydrochloride (GuHCl), suggesting that GuHCl is important for stabilizing viral RNA. PK6 was composed of 800 mM GuHCl, 400 μg/mL PK, 10% Tween 20 (T20) and 30 mM Tris-HCl pH 8.0 (Table 1).

**Figure 1:**
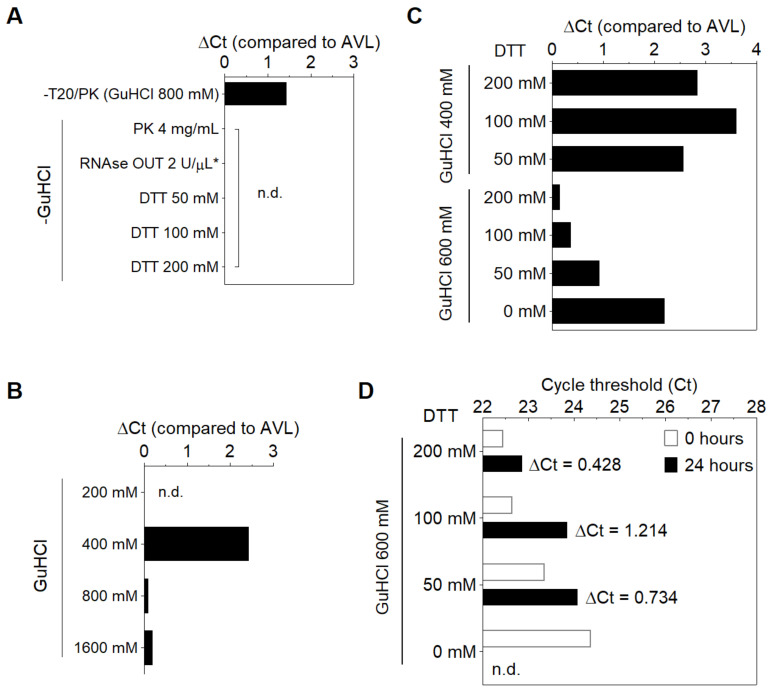
Modifications to the PK6 formula. (**A**) Removal of PK and T20 increases Ct value, while substituting GuHCl with PK (4 mg/mL), RNAse OUT or DTT results in no amplification. (**B**) 800 mM GuHCl is sufficient to stabilize RNA in the presence of PK and T20. (*) The solution containing RNAse OUT was buffered by Tris−EDTA pH 8.0 instead of Tris-HCl (6). (**C**,**D**) Optimization of the stabilization solution. (**C**) RNA stabilization is achieved by replacing PK and T20 with 200 mM DTT and reducing GuHCl to 600 mM, and (**D**) this is maintained after storage of processed specimens for 24 h at 8 °C. n.d. = not detected.

Aiming towards a minimal solution that enables RNA stabilization, several modifications were made to the PK6 formula to reduce the amount of components. The removal of T20 and PK increased the Ct value in relation to AVL (ΔCt = 1.433), while no amplification was detected when substituting GuHCl with PK at a higher concentration (4 mg/mL), RNAse OUT [11] or varying amounts of DTT (see below), further indicating that GuHCl is necessary to stabilize SARS-CoV-2 RNA in saliva (Figure 1A). By varying the amount of GuHCl in PK6, we observed that viral RNA could be detected only in concentrations above 400 mM, and that 800 mM was sufficient for RNA stabilization (ΔCt ≤ 0.104) in the presence of T20 and PK (Figure 1B). PK and T20 were then replaced with varying amounts of DTT (50 to 200 mM) in combination with lower concentrations of GuHCl (400 mM and 600 mM). Only the combination of 600 mM GuHCl and 200 mM DTT resulted in a negligible Ct increment in comparison to AVL (ΔCt = 0.148) (Figure 1C). Furthermore, saliva samples processed with this formulation presented the smallest Ct rise after storage at 8 °C for 24 h (ΔCt = 0.428) (Figure 1D). Together, these results indicate that heating saliva in this solution (30 mM Tris-HCl pH 8.0, 600 mM GuHCl, 200 mM DTT) at 65 °C/15 min plus 95 °C/2 min protects viral RNA from degradation. This DTT/GuHCl solution is hereafter referred to as ‘DGS’.

### 3.2. Compatibility of DGS with Direct RT-LAMP in Saliva

**Figure 2:**
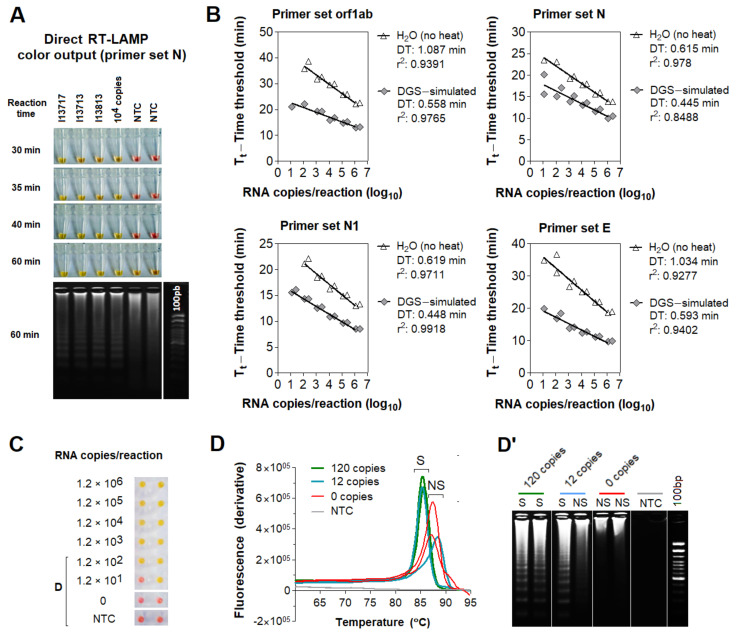
Effect of DGS heating on direct RT−LAMP reactions. (**A**) Compatibility between direct RT−LAMP and DGS−processed saliva. After 40−min incubation at 65 °C, colors were visibly distinguishable between the pink NTC and the yellow positive samples. Agarose gel electrophoresis confirmed specific amplification (as band patterns of individuals 13717, 13713 and 13813) matched those of the positive control (10^4^ RNA copies). Nonspecific amplification was observed in NTC after longer incubation (up to 60 min). (**B**–**D**) Compatibility with direct rtRT−LAMP. (**B**) SARS−CoV−2 RNA serially spiked in DGS−simulated saliva or in H_2_O was used to assemble standard curves for primer sets orf1ab, N, N1 and E, in duplicate reactions. Nonspecific and failed amplifications were observed at 0–120 copies/reaction in some reactions (not shown). Doubling time (DT) values were calculated to assess amplification speed/efficiency [12] for each primer set. Determination coefficients (r^2^) point to high linearity (>0.939) between RNA input and Tt in DGS−simulated saliva, except for primer set N (r^2^ = 0.8488). (**C**) Representative color output after rtRT−LAMP (primer set E is shown). (**D**) Representative dissociation analysis and (**D’**) gel electrophoresis showing non−specific LAMP products at 0 and 12 copies/reaction (primer set E is shown). NTC (no−template control) reactions were performed with H_2_O. S = specific amplification; NS = non−specific amplification.

Next, we examined whether DGS is compatible with direct RT-LAMP reactions. For this, we used saliva specimens from three individuals confirmed positive for COVID-19 via NOP swab RT-PCR by an external laboratory. In this test, specimens were heated with DGS under a slightly different protocol (55 °C/15 min followed by 95 °C/2 min) modified from previous work [8]. RT-LAMP was performed with the previously reported primer set N (Table S1) [9] and 10% volume of DGS:saliva (1:1) mix, resulting in a 20-fold dilution of the DGS formulation (Table 2). These three samples amplified specifically and changed color from pink to yellow within 30–40 min of reaction at 65 °C, while the no-template controls (NTCs) remained pink (Figure 2A), showing that DGS is compatible with colorimetric readouts.

To characterize the effects of the DGS heat treatment on RT-LAMP, we performed direct real-time RT-LAMP (rtRT-LAMP) with additional three published primer sets targeting different regions of the SARS-CoV-2 genome (orf1ab, N1, E; Table S1) [9,13,14]. Standard curves were made with serially diluted RNA spiked into DGS-processed saliva or H_2_O. Compared to RNA in H_2_O, DGS-simulated saliva showed higher reaction speed and amplification efficiency for all primer sets, revealed by reduction in time to threshold (Tt) and doubling time (DT) values, respectively (Figure 2B). DGS processing also enabled the formation of specific LAMP products and color change to yellow, in opposition to the nonspecific and failed amplifications, which remained pink (Figure 2C) and were clearly distinguishable from specific amplifications by dissociation analysis or agarose gel electrophoresis (Figure 2D,D’). Altogether, these results show that sample processing with DGS improves direct RT-LAMP reactions and allows SARS-CoV-2 detection either via endpoint analysis of color output or analysis of amplification and melting curves in rtRT-LAMP.

### 3.3. Optimization of the DGS Workflow

**Figure 3:**
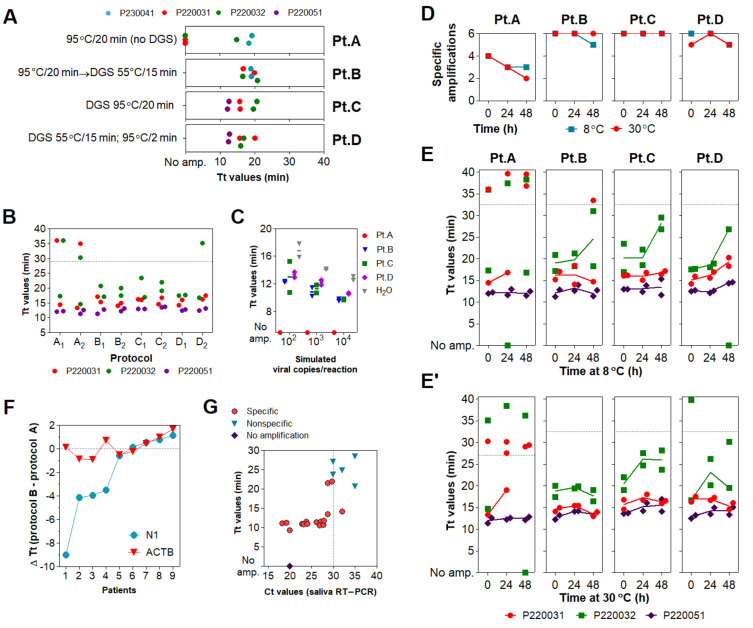
Assessment of different DGS heating protocols via rtRT−LAMP. (**A**) rtRT−LAMP results for clinical specimens heat−inactivated (no−DGS control) or processed under four DGS protocols (**A**–**D**). Reactions were performed on different days. (**B**) rtRT−LAMP results for clinical specimens after protocols (**A**–**D**), in two parallel experiments. Datapoints above the dashed line are nonspecific amplifications. rtRT−LAMP was performed in a single reaction plate. (**C**) Reaction output of simulated samples spiked with SARS−CoV−2 RNA (10^2^ to 10^4^ copies/reaction). RNAs spiked in H_2_O were used as positive control. Lines indicate mean Tt values. (**D**) Detection rate of the 3 DGS−processed clinical specimens incubated for 48 h at 8 °C and 30 °C (*n* = 6). (**E**,**E’**) rtRT−LAMP results after storage at 8 °C (**E**) and 30 °C (**E’**). Data points above the dashed line are nonspecific amplifications. Results shown in D/E/E’ were plotted with data from B (t = 0 h). (**F**,**G**) Assessment of protocol B in 11 additional specimens via rtRT−LAMP for primer sets N1 and *ACTB*. (**F**) Changes in reaction speed of protocol B compared to A were plotted as ΔTt values; negative values indicate gain in reaction speed. Nonspecific and failed amplifications were not included. (**G**) Saliva RT−PCR Ct values were plotted against Tt values used in (**F**). Experiments in (**A**–**E**) were performed with *n* = 3 biological samples (P220031, P220032, and P220041 or P220051). All rtRT−LAMP reactions were carried out in duplicates. Since GuHCl was recently shown to improve speed and sensitivity of RT−LAMP (13), all rtRT−LAMP performed on no−DGS controls (protocol A) were supplemented with 40 mM GuHCl to allow more precise evaluation of the DGS protocols.

The aforementioned protocols require mixing infected patient samples with DGS before viral inactivation, demanding high biosafety requirements. To overcome this issue, we sought to optimize sample processing to include a heat inactivation step before collection tubes were uncapped. We initially tested three saliva specimens heat-inactivated at 95 °C/20 min (protocol A) in the absence of DGS (no-DGS control), resulting in failed amplifications across reaction replicates in rtRT-LAMP. In contrast, subjecting these heat-inactivated specimens to a second heating step with DGS at 55 °C/15 min (protocol B) improves detection, leading to specific amplification in all replicates. This was also observed when specimens were mixed with DGS and single-heated at 95 °C/20 min (protocol C) or under the original protocol (55 °C/15 min; 95 °C/2 min; protocol D), suggesting that two-step heating (protocol B) does not overly affect stabilization of SARS-CoV-2 RNA in saliva (Figure 3A).

We further characterized DGS protocols B, C and D in comparison to the no-DGS control (protocol A). Three saliva specimens were processed under these protocols in two parallel experiments. rtRT-LAMP showed no differences in Tt values across protocols B, C, and D. However, we observed less specific amplification events under protocol A (four out of six replicates), while protocols B, C and D achieved specific detection in all replicates, except for one under protocol D (Figure 3B). Similarly, DGS protocols B, C and D enabled detection in simulated specimens (10^4^, 10^3^ and 10^2^ copies/reaction) without differences in the average Tt values, while no amplification was detected under protocol A, indicating that single heat inactivation in the absence of DGS is insufficient to counteract salivary ribonuclease activity (Figure 3C). To further compare RNA stabilization across protocols, the processed specimens (Figure 3B) were then incubated at 8 °C or 30 °C for up to 48 h. After 24 and 48 h of incubation at either temperature, the number of specific amplifications under protocol A further decreased, while greater detection rates (greater than or equal to five out of six replicates) were maintained with DGS protocols B, C and D (Figure 3D). Moreover, these three DGS protocols showed no appreciable shifts in average Tt values after 24 h at 8 °C (Figure 3E), while at 30 °C, this was only observed for protocol B (Figure 3E’). Together, these results suggest that the two-step protocol B is suitable to process clinical specimens, as it improves detection of SARS-CoV-2 compared to the no-DGS controls, and shows the most robust RNA stabilization effects.

To further assess its performance in rtRT-LAMP, protocol B was applied to 11 additional clinical samples and compared to single heat inactivation without DGS (protocol A). rtRT-LAMP was performed in duplicates with primer set N1 and the human-specific primer set *ACTB* (Table S1) [14]. In both methods, specific amplifications for *ACTB* were observed throughout all replicates, while specific amplifications for N1 were observed in at least one out of two replicates in nine samples. Differences in reaction speed (ΔTt) were calculated as average *Tt_Protocol B_*—*Tt_Protocol A_*. In four of these nine samples, an important reduction in Tt values was observed for protocol B, varying from 3.5 to 9 min, while *ACTB* showed no significant Tt fluctuations (Figure 3F). This indicates that protocol B improves detection rates (Figure 3A) and reaction speed (Figure 3F) in clinical saliva, and suggests that the latter effect may be specific to SARS-CoV-2 detection, as *ACTB* showed low ΔTt variability. Finally, viral RNA was extracted from these 11 specimens and RT-PCR Ct values were used to estimate viral load. We observed that SARS-CoV-2 RNA was detected in the majority of specimens with Ct < 30 (8/8), suggesting robustness for high viral loads (Figure 3G).

### 3.4. Optimization of Color Discrimination and Sample Processing Time

**Figure 4:**
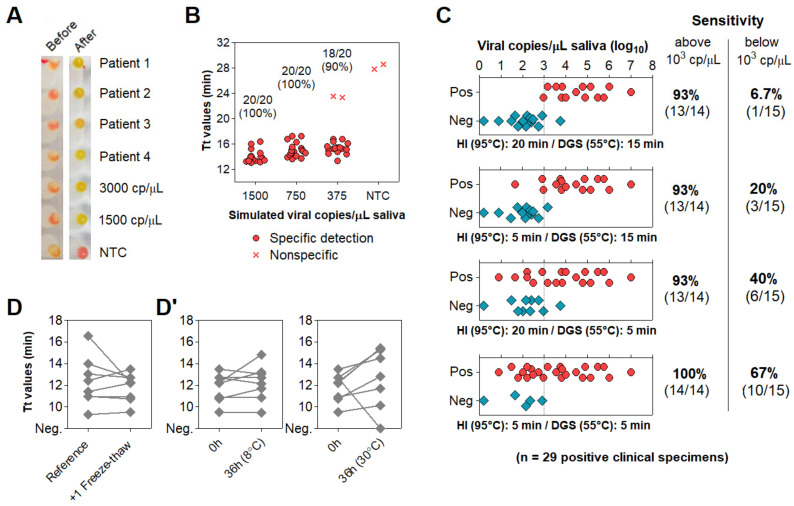
Direct rtRT−LAMP output after volumetric adjustments and reduction of processing time. (**A**,**B**) Output after volumetric adjustments. (**A**) Representative color output before and after rtRT−LAMP performed on 4 clinical specimens and on 3000 and 1500 simulated viral copies/µL. (**B**) Sensitivity using simulated specimens (20 replicates each). No−template control (NTC) reactions had H_2_O as input. (**C**) Reduction of heat inactivation (HI) and DGS incubation times with respective rtRT−LAMP readout and sensitivity (stratified at 10^3^ copies/μL; dashed line). (**D**,**D’**) Before−after plots for 8 specimens processed with HI for 5 min and DGS for 5 min. (**D**) rtRT−LAMP Tt profiles after 1 freeze–thaw cycle. (**D’**) Tt profiles after storage for 36 h at 8 °C and 30 °C. cp/μL = viral copies per μL of saliva.

We observed that up to 15% of specimens showed discordant color output and amplification results after RT-LAMP followed by agarose gel electrophoresis. This could be explained by pH variation of saliva or excess of salivary inhibitors impairing amplification efficiency. To ensure appropriate color-based analysis, volumetric adjustments were implemented in the DGS and RT-LAMP protocols. The DGS:saliva ratio was increased to 2:1 to enhance inactivation of salivary nucleases by DTT/GuHCl, while sample input into RT-LAMP reactions decreased from 1.25 µL to 1 µL and the volume of Colorimetric Master Mix increased from 6.25 µL to 8.25 µL (Table 3). These modifications resulted in better color discrimination after RT-LAMP, as color change and viral RNA amplification showed no discrepancies (Figure 4A). These adjustments were associated with a >95% limit of detection at 750 viral copies/µL in simulated specimens (Figure 4B).

To accelerate sample processing, we tested saliva samples confirmed positive via RT-PCR, as better detailed in the next item. We simultaneously processed 29 positive specimens with the previously devised protocol (heat inactivation for 20 min at 95 °C and DGS stabilization for 15 min at 55 °C) and under shorter processing times (5 min at 95 °C and 5 min at 55 °C). Here, primer set N1 was replaced by E1, which is more sensitive [15] (Table S1). Shortening both heat inactivation and DGS stabilization to 5 min led to the largest improvement in detection rate, enabling detection in 100% (14/14) of samples containing over 10^3^ viral copies/μL and 67% (10/15) of samples below that cutoff, a 10-fold improvement over the unmodified protocol (Figure 4C). These results show that reducing sample processing time improves direct RT-LAMP sensitivity, particularly for low viral loads in saliva.

To assess conservation of diagnostic properties, 8 of these DGS:saliva mixtures were stored at −80 °C, thawed and analyzed via direct rtRT-LAMP, without consistent increments in Tt values. Considering that the processed specimens were freeze–thawed twice before this test, this suggests that DGS-processed saliva withstand repeated freeze–thaw cycles (Figure 4D). Following this, DGS:saliva mixtures were incubated at 8 °C and 30 °C for 36 h. At 8 °C, no consistent Tt increments were observed, while at 30 °C, all samples showed increased Tt values, and one returned negative, suggesting that refrigeration is necessary to maintain the diagnostic properties of specimens after processing (Figure 4D’).

### 3.5. Characterization of the Diagnostic Properties of Saliva Compared to NOP Swabs

**Figure 5:**
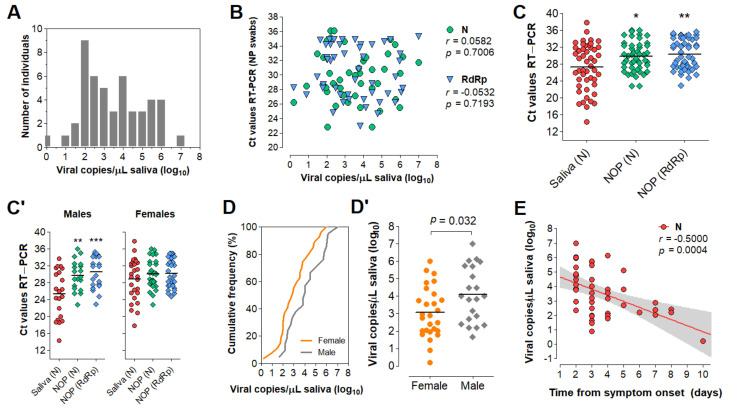

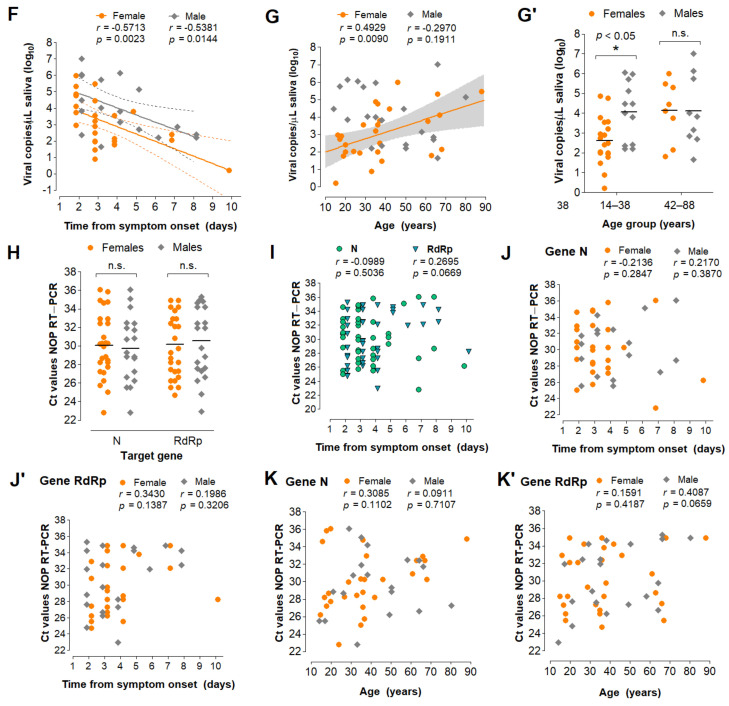
Analysis of saliva as a diagnostic specimen for SARS−CoV−2 detection via direct RT−LAMP. (**A**) Distribution of viral load in saliva from 51 COVID−19 patients. (**B**) Pearson’s correlation between viral load in saliva (gene N) and Ct values of NOP swab specimens (genes N and RdRp). (**C**) Comparison of mean Ct values between saliva and NOP swab specimens; and (**C’**) the same analysis was performed for each gender. One−way ANOVA with Dunnet’s post−tests; * *p* < 0.05; ** *p* < 0.001. (**D**) Cumulative frequency distribution and (**D’**) comparison of mean viral loads between males and females; Student’s *t*-test. (**E**) Pearson’s correlation between salivary viral load and days since onset of symptoms and (**F**) the same analysis stratified by gender. Dashed lines or gray shading indicate 95% confidence intervals of the linear trends for significant correlations. (**G**) Pearson’s correlation between viral load and age for each gender. (**G’**) Comparison of mean viral loads between genders for younger (aged 14–38) and older (aged 42–88) individuals; Two−way ANOVA with Bonferroni post−tests. (**H**–**K’**) Comparisons performed for saliva (**D’**–**G**) were applied to NOP swabs using Ct values from RT−PCR targeting N and RdRp (**H**–**K’**).

To evaluate the diagnostic performance of saliva, we analyzed 51 saliva samples collected concomitantly with NOP swabs from symptomatic individuals upon hospital admission. These cases were confirmed by an external laboratory via NOP RT-PCR targeting genes N and RdRp. We quantified viral copies in the paired saliva specimens via RT-PCR for the N gene, wherein 48 specimens returned positive (94% agreement). These samples showed viral loads between 1.6 and 10^7^ copies/µL of saliva (0.2–7 log_10_ copies/µL; mean = 3.51, SD = 1.51) (Figure 5A; Table S2). No correlation between NOP swab Ct values and viral copies in saliva was observed (Figure 5B). On average, the Ct values in saliva were lower compared to NOP swabs, indicating higher viral load in saliva (Figure 5C).

Next, we analyzed viral load stratified by gender, time from onset of symptoms, and age (Table S2). These clinical data were available for 49 of the 51 individuals. Interestingly, males showed lower mean Ct values in saliva compared to NOP swabs, which was not observed for females (Figure 5C’). Furthermore, males showed significantly higher viral loads in saliva than females (mean difference = 1.03 log_10_ copies/µL; *p* = 0.032, Student’s *t*-test) (Figure 5D,D’), while no gender differences were found for NOP Ct values. In both male and female saliva, viral load peaked during the initial 2 days of symptoms and was negatively correlated with time (range = 2 to 10 days from onset of symptoms) (Figure 5E). Linear regression analysis showed that salivary viral load falls below 10^3^ copies/µL at around 5 days after symptom onset (x = 4.92 days [95% CI: 3.92–6.86]), which in females occurs earlier than in males (x_females_ = 3.74 days [95% CI: 2.42–5.24]; x_males_ = 6.35 days [95%CI: 4.71–15.38]) (Figure 5F). Further, viral load in saliva was positively correlated with age in females, but not in males (range = 14–88 years) (Figure 5G). Notably, among younger patients (aged 14–38), females showed lower viral load than males, while no differences were observed in older individuals (aged 42–88), suggesting that the observed gender differences in salivary viral load decrease with age (Figure 5G’). In NOP swabs, no relationship was detected between viral load (Ct values) and age, gender or days since symptom onset (Figure 5H–K). Together, these results show that viral load in saliva peaks in the early days of symptoms and is depleted with time, with females displaying lower viral load compared to males.

### 3.6. Direct RT-LAMP in 80 Saliva Samples from Symptomatic Individuals

Without prior knowledge of NOP RT-PCR results, we tested 80 additional saliva samples from symptomatic individuals collected concomitantly with NOP swabs. Specimens were processed with the established DGS protocol. Direct rtRT-LAMP was carried out with primer sets N1, E1 and As1e, which was also reported to be more sensitive than N1 (Table S1) [15], in addition to the human-specific primer set *ACTB*. All specimens showed successful amplification for *ACTB*, confirming adequate sample quality. In preliminary analyses, we observed that E1 was more sensitive than N1 and As1e, so reactions using E1 were performed in duplicates to increase sensitivity and to minimize false negatives. Results were considered positive upon detection in at least one of them, while single reactions were carried out with N1 and As1e. Nonspecific and failed amplifications were considered negative. RT-PCR in NOP specimens reported 51 positives (NOP^+^) and 29 negatives (NOP^−^).

**Figure 6:**
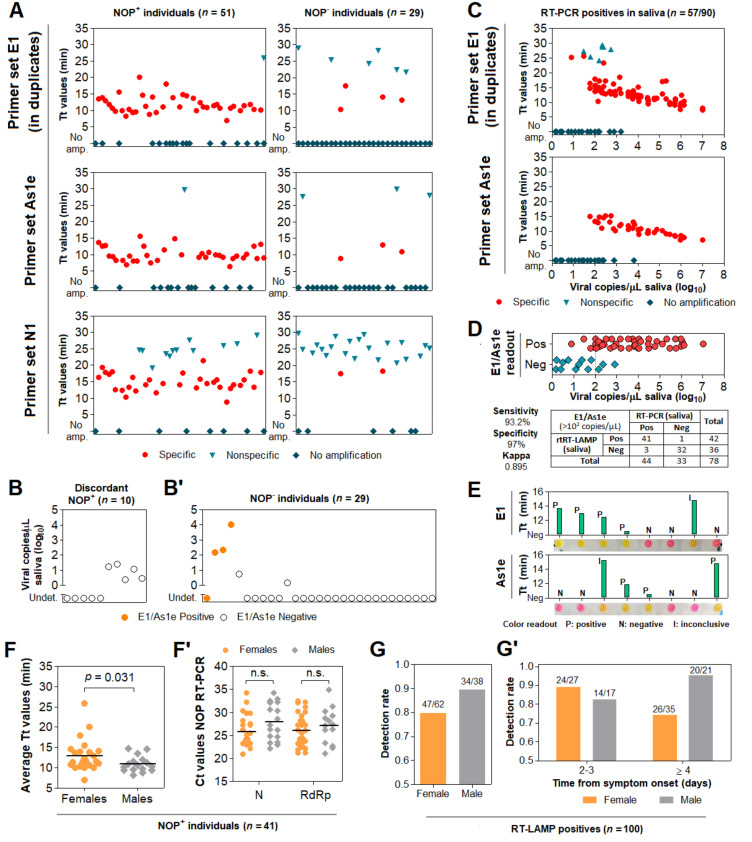
Assessment of the DGS and direct RT-LAMP diagnostic workflow in clinical saliva. (**A**) Saliva direct rtRT-LAMP output in NOP^+^ (*n* = 51) and NOP^-^ (*n* = 29) individuals with primer sets E1 (in duplicates), As1e and N1. Specific amplifications were classified as positive detections, while nonspecific and failed amplifications were classified as negative. (**B**) RT-PCR quantification of viral copies in saliva from the 10 discordant NOP^+^ individuals who escaped detection via rtRT-LAMP and (**B’**) in saliva from the 29 NOP^−^ individuals, including the 4 discordant rtRT-LAMP positives. Undet. = undetermined. (**C**) rtRT-LAMP with primer sets E1 (in duplicates) and As1e for saliva specimens in which viral titer was determined via RT-PCR (shown in B/B’ and in Figure 5). (**D**) Assessment of sensitivity, specificity and Kappa values of rtRT-LAMP considering primer set E1 alone or E1/As1e for specimens containing at least 10^2^ viral copies/µL (dashed line). (**E**) Representative comparisons between visual color interpretation and rtRT-LAMP results (Tt values). Over 97.6% of specimens were correctly classified as positive (P, yellow) or negative (N, pink) for E1 and As1e, and the remaining was classified as inconclusive (I, orange-shaded). (**F**) Gender comparison of average Tt values in saliva from the NOP+ cases ascertained in ‘A’ (*n* = 41 rtRT-LAMP positives). Results for primer set E1 were used. Mann–Whitney test was used to compare medians. (**F’**) Gender comparison of Ct values from NOP RT-PCR for genes N and RdRp. Student’s *t*-test was used to compare means. n.s. = not significant. (**G**) Comparison of joint E1/As1e detection rates between genders for 100 saliva specimens ascertained herein via rtRT-LAMP. (**G’**) Stratified analysis of detection rates between genders, based on days from onset of symptoms.

Among the 51 NOP^+^ individuals, saliva rtRT-LAMP returned positive in 40 with primer set E1, 35 with primer set As1e, and 31 with primer set N1, which was excluded from subsequent analyses due to reduced sensitivity and excessive nonspecific amplifications (Figure 6A). Joint analysis considering either E1 or As1e resulted in 41 positives and 10 negatives. RT-PCR on saliva from these 10 discordant NOP^+^ individuals revealed either viral loads below 10^2^ copies/µL or absence of SARS-CoV-2 (Figure 6B).

Among the 29 NOP^−^ individuals, joint E1/As1e analysis returned four positive saliva specimens, which were confirmed in an independent rtRT-LAMP assay. Of note, RT-PCR on saliva from these NOP^−^ individuals detected SARS-CoV-2 at >10^2^ copies/µL in 3 of the 4 E1/As1e positives (~6%; 3/51). The remaining patients, negative in both NOP RT-PCR and saliva rtRT-LAMP, showed either undetectable (23/25) or low salivary viral load (<10^1^ copies/µL; 2/25) (Figure 6B’). These results further highlight viral load disparities between NOP and saliva specimens, and indicate that a fraction of infected patients escape detection via NOP RT-PCR.

### 3.7. Clinical Performance of the Direct RT-LAMP Workflow

To evaluate the diagnostic capabilities of our workflow, we tested a total of 91 saliva specimens in which viral load was herein determined via RT-PCR, comprising 58 specimens confirmed positive and 33 specimens confirmed negative for SARS-CoV-2. These specimens had been processed with DGS and stored at −80 °C. rtRT-LAMP reactions were carried out using E1 in duplicates and As1e in single reactions, as previously described. One positive sample failed to amplify *ACTB*, likely due to low quality, and was excluded from the analysis. rtRT-LAMP correctly identified 38/57 positive specimens with As1e and 44/57 in at least one of the E1 replicates, with the majority of specimens that escaped detection displaying viral loads below 10^2^ copies/µL (Figure 6C). Notably, since all 38 As1e-positives were covered by E1, joint E1/As1e analysis also returned 44/57 positives, suggesting little benefit from pairing E1 and As1e as presented. Among the 33 saliva specimens negative for SARS-CoV-2, we observed one positive result by rtRT-LAMP. Considering all 90 specimens and the full range of viral loads represented here, this results in 77.2% overall sensitivity and 97% specificity with primer set E1 or joint E1/As1e. Importantly, stratification at 10^2^ copies/µL resulted in 93.2% sensitivity (41/44 positives) and Kappa = 0.895 for viral loads above this cutoff, demonstrating the high sensitivity, specificity and reliability of the test (Figure 6D).

Next, we sought to determine the efficiency of the RT-LAMP color readout upon visual examination. Without prior knowledge of the results, the color output of 131 samples tested herein via rtRT-LAMP was visually classified as positive (yellow), negative (pink) or inconclusive (orange-shaded), for primer sets E1 and As1e. All yellow- and pink-colored reactions were correctly classified as positive and negative, respectively, resulting in 97.6% agreement for primer set E1 (206/211 reactions) and 97.7% agreement for primer set As1e (128/131 reactions). The orange-shaded output of the remaining reactions resulted from nonspecific or specific amplifications, mostly with late Tt values in rtRT-LAMP, indicating the absence of SARS-CoV-2 or low viral loads, respectively (Figure 6E). This demonstrates high agreement between endpoint color output and rtRT-LAMP analysis.

Finally, to confirm our findings regarding viral load differences between males and females, we analyzed E1 Tt values from rtRT-LAMP performed on saliva from the above NOP+ cases (*n* = 41 out of 51). Albeit less sensitive than RT-PCR, this approach evidenced lower Tt values in male saliva, indicating higher viral load (Figure 6F), while no differences were observed between NOP RT-PCR Ct values (Figure 6F’). Moreover, analysis of rtRT-LAMP results from 100 cases ascertained herein (Table S3) revealed a lower detection rate in women compared to men (79.7% and 89.5%, respectively; Figure 6G). Stratification based on days from onset of symptoms showed increased detection rate in women tested up to the third day (88.9%), and decreased detection in women tested from the fourth day onward (74.3%). Compared to females, males consistently show high detection rates up to the third day and beyond (82.4% and 95.3%, respectively). These observations are in line with females showing lower viral load and faster viral clearance in saliva compared to males, which may adversely impact salivary diagnostics (Figure 6G’).

## 4. Discussion

In this work, we developed a DTT/GuHCl-based RNA stabilization solution (DGS) and a workflow that enables the robust detection of SARS-CoV-2 in saliva via direct RT-LAMP, with either colorimetric or real-time fluorescence readout. We showed that heat treatment of saliva mixed with DGS protects SARS-CoV-2 RNA from degradation, while improving efficiency, reaction speed and detection rates during subsequent analysis by RT-LAMP. We also characterized saliva and NOP swab specimens according to viral load, gender, age, and time from symptom onset, providing more insight into the advantages and limitations of SARS-CoV-2 salivary diagnostics.

In simulated saliva, we observed that heat treatment with PK-based formulations is insufficient to stabilize viral RNA, contrasting with previous reports using samples containing SARS-CoV-2 virions [8,16,17,18]. This discrepancy could be explained by the fact that the spiked RNA in our simulated samples is not protected by the nucleocapsid and other structural viral proteins, being readily digested by any active nucleases left after sample processing. This indicates that PK did not sufficiently inactivate salivary nucleases under the conditions examined here. Thus, care must be taken when using PK and other agents to process specimens, especially when combined with additional viral lysis methods, which may expose viral RNA to digestion. Accordingly, although SARS-CoV-2 remains stable in crude saliva [19], diagnostic sensitivity may drop depending on the method employed to inactivate/process samples, such as heat inactivation, inclusion of detergents, and other factors [20,21], making the RNA protection provided by DGS nevertheless critical.

In clinical saliva, DGS heat treatment improves detection of SARS-CoV-2 compared to heating without DGS. The active ingredients in DGS act by reducing and denaturing salivary extracellular ribonucleases and other inhibitors, and may also facilitate access of RT-LAMP enzymes to the SARS-CoV-2 genome through reduction and denaturation of viral proteins. GuHCl was recently reported to improve speed and sensitivity when added to RT-LAMP [14], so the carryover of DGS into reactions may cooperate to increase SARS-CoV-2 detection by modulating the reaction chemistry as well. Importantly, this carryover is not solely responsible for the rise in detection rates and reaction speed in clinical specimens, because these effects were observed in relation to no-DGS controls in which RT-LAMP was supplemented with GuHCl (40 mM). Thus, DGS improves SARS-CoV-2 detection both via stabilization of the viral genome and increasing RT-LAMP speed/sensitivity. Furthermore, DTT/GuHCl are also mucolytic agents [22], and therefore ameliorate the pipetting of viscous specimens.

Of the DGS protocols investigated here, the best overall performance was achieved with a two-step method that includes heat inactivation of the saliva in the collection vessel before further manipulation (protocol B). This protocol improved direct RT-LAMP speed and detection rates compared to the no-DGS control, and elicited the strongest RNA stabilization effects compared to the other methods we examined. The volumetric adjustments to the DGS:saliva ratio and RT-LAMP mastermix improved endpoint color interpretation, while shortening the heat inactivation and DGS steps to 5 min each improved sensitivity for specimens containing less than 10^3^ copies/µL, likely owing to greater RNA stability. These steps should suffice to inactivate SARS-CoV-2, since complete viral inactivation has been reported for periods as short as 3 min at 95 °C [23,24]. Furthermore, the final sample processing protocol is amenable to repeated freeze–thaw cycles and allows storage at 8 °C for up to 36 h without adversely affecting diagnostic output, which is important if RT-LAMP must be performed or repeated later. Finally, shortening sample processing further reduced diagnostic turnaround time. This two-step protocol reduces the risk of infection by healthcare specialists and clinicians since the collection vessel remains sealed until heat inactivation of the virus, thus facilitating diagnostic workflows and alleviating biosafety concerns in point-of-care settings and in test sites lacking in sophisticated infrastructure.

After these modifications, RT-LAMP achieved 77.2% overall clinical sensitivity and 97% specificity, with 93.2% sensitivity in saliva containing at least 10^2^ viral copies/µL (Kappa = 0.895), which is on par or more sensitive compared to most of the direct RT-LAMP approaches reported so far [8,15,25,26,27,28,29,30,31,32,33]. Moreover, the high agreement (>97.6%) observed between color interpretation and specific amplification plots in rtRT-LAMP demonstrates reliability on end-point color readout if real-time analysis is not possible. Although only 23% of specimens containing less than 10^2^ viral copies/µL were detected by the present method, the gain in exam turnaround time may be desirable at the cost of sensitivity, since lower viral loads are associated with lower transmissibility of SARS-CoV-2 [34]. Likewise, it has been proposed that effective COVID-19 surveillance depends on test frequency and turnaround time rather than on test sensitivity [35], especially for identifying nonsymptomatic carriers. Therefore, periodic testing using a faster, cheaper and noninvasive saliva protocol may be preferable to more lengthy and costly methods, such as standard RT-PCR workflows. Still, if test sensitivity poses an issue in SARS-CoV-2 detection efficiency, this may be mitigated if subjects are tested when salivary viral load is highest, as discussed below.

Using RT-PCR, we detected SARS-CoV-2 in 94% of the saliva specimens from symptomatic patients, with Ct comparisons suggesting that viral load in saliva may be higher than in NOP specimens, particularly in males. The observed absence of correlation between NOP and salivary viral loads reflects distinct viral shedding dynamics in these tissues [2]. Our results confirm that saliva is a suitable diagnostic specimen compared to NOP swabs, especially in cases where SARS-CoV-2 is detectable only in saliva, as ascertained in three patients herein. Male sex and old age are risk factors for developing severe COVID-19 [36,37]. Here, males showed mean viral load around 10 times higher than females in saliva but not in NOP swabs, which was confirmed in a second cohort with the use of RT-LAMP Tt values to estimate viral load. Since airborne transmission is related to the amount of virions per saliva droplet, this indicates that males could be more likely to spread the virus than females. Notably, compared to age-matched males, young females (<38 years old) showed lower salivary viral load that increases with age, while no clear age-related effects were found in males. Clinical severity and immunological profiles seem to better correlate with viral load in saliva than in NOP swabs [38,39], so our findings could be attributed to distinct immune responses between genders leading to higher viral shedding and disease severity in males [40], and could also explain the higher proportion of asymptomatic females in couples positive for SARS-CoV-2 [41]. These observations further suggest that salivary viral load together with older age, male gender and other risk factors could be important to predict disease duration, severity and mortality [39,42]. Contrasting the lack of correlation between viral load and time in NOP swabs, viral load in saliva peaked in the first days of symptoms and declined within 10 days, agreeing with recent estimates that show peak transmissibility 1.8 days before the onset of symptoms and low chance of transmission beyond 9.5 days after onset [43]. Importantly, we estimate that the sensitivity threshold of 10^3^ copies/µL employed by several RT-LAMP approaches is crossed around the 5th day of symptoms, with males showing delayed virus clearance compared to females. Accordingly, we observed a lower detection rate via RT-LAMP in females on the 4th day of symptoms and beyond, compared to females tested earlier. Based on these observations, we suggest that salivary diagnostics in symptomatic individuals should be performed as soon as possible after symptom onset to increase chances of detection and to overcome limitations in sensitivity, especially for females.

In summary, we report a simple and rapid RT-LAMP diagnostic workflow that obviates RNA extraction and specialized equipment, providing a cost- and time-efficient alternative to standard RT-PCR diagnostics. The use of DGS to process specimens and modifications to the RT-LAMP reaction resulted in high sensitivity and specificity to detect SARS-CoV-2 in saliva via real-time or endpoint colorimetric analysis, thus qualifying it for point-of-care testing. This work not only provides recommendations to optimize viral detection, but it also indicates that saliva may provide clues to clarify the biological determinants of SARS-CoV-2 infection and transmission. Recent reports show that saliva has higher sensitivity than nasal/nasopharyngeal swabs for identifying asymptomatic cases [41], and that viral load distribution is equivalent in saliva from symptomatic and nonsymptomatic individuals [42]. Considering that a fraction of infected patients escape detection via NOP RT-PCR, and since saliva better correlates with transmissibility and clinical outcomes, rapid salivary diagnostics stands as an invaluable opportunity to efficiently tackle the COVID-19 pandemic. We reinforce that rapid saliva tests should be prioritized in screenings to suppress viral spread, which may be extended to other respiratory pathogens.

## Figures and Tables

**Table 1:** Solutions used in the initial screening and Ct values after processing.

	Composition	Ct
AVL	Guanidinium thiocyanate (50–70%)	23.15
PK1	PK (2 mg/mL)	ND
PK2	PK (0.4 mg/mL)	ND
PK3	PK (0.4 mg/mL) + T20 (10%)	ND
PK4	PK (0.4 mg/mL) + T20 (10%) + Tris-HCl pH 8.0 (30 mM)	ND
PK5	PK (0.4 mg/mL) + Tris-HCl pH 8.0 (30 mM)	ND
PK6	PK (0.4 mg/mL) + T20 (10%) + GuHCl (800 mM) + Tris-HCl pH 8.0 (30 mM)	23.9

PK: Proteinase K; T20: Tween 20; GuHCl: guanidine hydrochloride; ND: not detected.

**Table 2:** DGS constituents and carryover into RT-LAMP.

Component	DGS	DGS:Saliva (1:1)	Carryover into RT-LAMP (1.25 µL Input)
GuHCl	600 mM	300 mM	30 mM
DTT	200 mM	100 mM	10 mM
Tris-HCl pH 8.0	30 mM	15 mM	1.5 mM
Saliva	-	100 µL	0.625 µL
Total volume	100 µL	200 µL	12.5 µL

**Table 3:** DGS constituents and carryover into RT-LAMP after volumetric adjustments.

Component	DGS	DGS:Saliva (2:1)	Carryover into RT-LAMP (1 µL Input)
GuHCl	600 mM	400 mM	32 mM
DTT	200 mM	133.3 mM	10.66 mM
Tris-HCl pH 8.0	30 mM	20 mM	1.6 mM
Saliva	-	100 µL	0.33 µL
Total volume	200 µL	300 µL	12.5 µL

## Data Availability

The data presented in this study are available on request from the corresponding authors. The data are not publicly available due to privacy restrictions.

## Supplementary Materials

The following are available online at https://www.mdpi.com/article/10.3390/diagnostics11081400/s1, Table S1: Primer set components used in this work, Table S2: Clinical information and RT-PCR results in NOP swabs and saliva, Table S3: Clinical information and direct RT-LAMP results.
